# Neonatal tobacco smoke reduces thermogenesis capacity in brown adipose tissue in adult rats

**DOI:** 10.1590/1414-431X20186982

**Published:** 2018-04-19

**Authors:** T.C. Peixoto, E.G. Moura, E. Oliveira, V. Younes-Rapozo, P.N. Soares, V.S.T. Rodrigues, T.R. Santos, N. Peixoto-Silva, J.C. Carvalho, C. Calvino, E.P.S. Conceição, D.S. Guarda, S. Claudio-Neto, A.C. Manhães, P.C. Lisboa

**Affiliations:** 1 Laboratório de Fisiologia Endócrina, Instituto de Biologia, Universidade do Estado do Rio de Janeiro, Rio de Janeiro, RJ, Brasil; 2 Laboratório de Neurofisiologia, Instituto de Biologia, Universidade do Estado do Rio de Janeiro, Rio de Janeiro, RJ, Brasil

**Keywords:** Tobacco smoke, Suckling period, Developmental plasticity, Obesity, Autonomic function

## Abstract

Maternal smoking is a risk factor for progeny obesity. We have previously shown, in a rat model of neonatal tobacco smoke exposure, a mild increase in food intake and a considerable increase in visceral adiposity in the adult offspring. Males also had secondary hyperthyroidism, while females had only higher T4. Since brown adipose tissue (BAT) hypofunction is related to obesity, here we tested the hypothesis that higher levels of thyroid hormones are not functional in BAT, suggesting a lower metabolic rate. We evaluated autonomic nerve activity in BAT and its function in adult rats that were exposed to tobacco smoke during lactation. At birth, litters were adjusted to 3 male and 3 female pups/litter. From postnatal day (PND) 3 to 21, Wistar lactating rats and their pups were divided into SE group, smoke-exposed in a cigarette smoking machine (4 times/day) and C group, exposed to filtered air. Offspring were sacrificed at PND180. Adult SE rats of both genders had lower interscapular BAT autonomic nervous system activity, with higher BAT mass but no change in morphology. BAT UCP1 and CPT1a protein levels were decreased in the SE groups of both genders. Male SE rats had lower β3-AR, TRα1, and TRβ1 expression while females showed lower PGC1α expression. BAT Dio2 mRNA and hypothalamic POMC and MC4R levels were similar between groups. Hypothalamic pAMPK level was higher in SE males and lower in SE females. Thus, neonatal cigarette smoke exposure induces lower BAT thermogenic capacity, which can be obesogenic at adulthood.

## Introduction

The “developmental origins of health and disease” (DOHaD) concept relates the influence of disturbances during a critical window of development on the induction of permanent changes that determine a pattern of health or disease later in life (1). Evidence indicates that environmental and nutritional changes during periods of great plasticity (intrauterine life and/or lactation) induce metabolic disorders, for instance obesity, in the offspring. This phenomenon is known as metabolic programming or developmental plasticity (2,3).

Exposure to cigarette smoke during gestation and lactation can lead to developmental plasticity (4). Epidemiological data showed that maternal smoking is a risk factor for obesity in the progeny (5). Nicotine, the main psychoactive compound of the cigarette smoke, acts as an “endocrine disruptor” during suckling, promoting epigenetic changes that result in altered metabolic patterns at adulthood (6). Our research group has been studying the effect of tobacco smoke exposure exclusively during lactation and we have demonstrated that this insult can cause obesity development and endocrine disorders in the adult offspring (7,8). In this model, adult offspring have higher total body fat and visceral fat despite a mild hyperphagia, suggesting that they are hypometabolic. Surprisingly, males show secondary hyperthyroidism (higher TSH, T4, and T3) and increased adrenal catecholamines content (7), which apparently suggest a hypermetabolic status, whereas females show only higher serum T4 without presenting changes regarding catecholamines (9). All these changes are capable of altering the brown adipose tissue (BAT) thermogenesis (10).

BAT hypofunction is related to obesity since it regulates body temperature and energy expenditure. This type of adipocyte has a large number of mitochondria rich in uncoupling protein 1 (UCP1) (11). UCP1 is capable of decoupling the mechanism of oxidative phosphorylation from the respiratory chain, producing heat and thereby increasing thermogenesis (12). Non-shivering thermogenesis is regulated mainly by sympathetic nervous system and thyroid hormones (TH). Thus, beta 3-adrenergic receptor (β3-AR) (13) and the TH receptors TRβ1 and TRα1 (14) are considered BAT biomarkers. Type 2 iodothyronine deiodinase (Dio2) is responsible for local T4 to T3 conversion and is regulated by adrenergic stimulation (15). Other mediators act increasing or decreasing thermogenesis, such as the peroxisome proliferator-activated receptor-coactivator (PGC1α) that regulates the mitochondrial biogenesis and respiratory function (16), and the carnitine palmitoyltransferase 1A (CPT1a), which controls the fatty acid oxidation (17). Recently, Fan et al. showed, in a model of maternal prenatal and lactation nicotine exposure, morphological changes in the BAT, such as lower number of mitochondria and a whitening phenotype associated with lower expression of PGC-1α and UCP1, characterizing BAT hypofunction in the adult male rat offspring (18).

Energy balance also involves a complex network of central nervous system communication with peripheral tissues. The hypothalamus participates in the control of food intake and energy expenditure, regulating BAT thermogenesis (19). An important region in this control is the ventromedial nucleus of the hypothalamus (VMH), whose actions are mediated via the AMP-activated protein kinase (AMPK), which decreases the thermogenesis (20). The AMPK-mediated thermogenesis is mainly inhibited by thyroid hormones of the VMH (21). Another central regulator of BAT thermogenesis is the melanocortin system, which has neural projections stimulating the sympathetic nervous activity of BAT, improving thermogenesis (22). The alpha-melanocyte-stimulating hormone (α-MSH) produced in the arcuate nucleus (ARC) is derived from the reparaventricular nucleus (PVN).

As mentioned, we have characterized a gender dimorphism in thyroid and adrenal function of rats programmed by neonatal tobacco smoke exposure (7–9). Although males have higher TH and females have higher T4, they may have TH resistance in BAT. We hypothesized that the exposure to cigarette smoke during lactation can induce BAT hypofunction at adulthood, promoting fat accumulation in both genders. Thus, we investigated the long-term repercussions of tobacco smoke exposure during lactation on: 1) BAT sympathetic nerve activity, 2) BAT morphology, 3) BAT biomarkers of catecholamine and TH sensitivity (UCP1, β3-AR, TRα1, TRβ1, and Dio2), mitochondrial biogenesis (PGC1α) and fatty acid oxidation (CPT1a), and 4) hypothalamic regulators of BAT thermogenesis (AMPK, POMC, and MC4R).

## Material and Methods

The Animal Care and Use Committee of the Biology Institute of the Universidade Estadual do Rio de Janeiro approved our experimental design (CEUA/019/2014). In accordance with the Brazilian Law No. 11.794/2008, experiments were done to minimize the number of rats and the suffering caused by the experimental procedures, following the ethical guideline of the three “Rs” - reduction, refinement, and replacement.

### Model of direct tobacco smoke exposure during lactation

**Figure 1.**
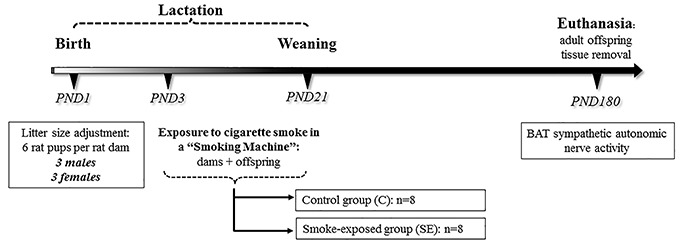
Experimental timeline. PND: postnatal day; BAT: brown adipose tissue.

Wistar rats were housed under controlled temperature (23±1°C), photoperiod of 12 h (light/dark cycle), and free access to water and food. For mating, twenty adult female rats (200–225 g) were placed with ten adult male rats (300–325 g) for 5 days. After this, sixteen pregnant rats were housed in individual cages until delivery. After birth, which was considered as the first postnatal day (PND1), all litters were adjusted to 6 pups for each dam (3 females and 3 males). As depicted in Figure 1, three days after birth, lactating dams and their offspring were randomly divided into two groups: smoke-exposed (SE, n=8) and control (C, n=8).

The SE group was directly exposed throughout lactation (until PND21) to tobacco smoke in a cigarette-smoking machine (TE-10, Teague Enterprises, USA), 4 times per day (1 h each exposure), as previously reported (7,8). This machine generated tobacco smoke from type 3R4F research cigarettes (nicotine=0.73 mg/cigarette; Reference Cigarette Program, University of Kentucky, USA). The levels of the main nicotine metabolite, cotinine, have previously been measured in the dams’ serum and milk as well as in the offsprings’ serum in our model (7,8) and represent the cotinine levels detected in heavy smokers (23). The C group was exposed for the same period to ambient air in a chamber similar to the one used for the SE-group exposure.

Weaning occurred at PND21 and then male and female pups were kept in different boxes (3 males and 3 females) until PND180. From PND21 to PND180 (euthanasia day), food intake and body mass were evaluated every 4 days. The estrous cycle was analyzed after PND150. Females were euthanized during diestrous. SE and C females had regular 4- to 5-day estrous cycles, indicating that the programming by tobacco smoke exposure does not affect the reproductive cycle (9).

### Autonomic nervous system (ANS) activity on interscapular brown adipose tissue (iBAT)

At PND180, one animal from each litter per group (C and SE) was fasted for 12 h and then anesthetized (ketamine 70 mg/kg and xylazine 7 mg/kg) for *in vivo* autonomic nerve electrical activity assessment as previously described (24). Interscapular BAT sympathetic nerve activity (SNA) from the left interscapular nerve was exposed under a dissection microscope. The branches were placed on a pair of hook platinum electrodes connected to an electronic device (Bio-Amplificator, Insight¯, Brazil) to record the electrical signals. In order to avoid dehydration, the nerve was covered with mineral oil. Nerve activity was amplified (10,000×) and filtered (cut-off: 60 kHz). Results were analyzed using the PowerLab data acquisition system (8SP; AD Instruments, Australia). All nerve activity recordings were carried out inside a Faraday cage to avoid electromagnetic interference. Animals were kept under a warming light. After 10 min of stabilization, the average number of spikes per 10 s intervals during a 10-min period was calculated (24). The background noise level was determined in a nerve segment.

### Euthanasia and tissue collection

After the measurement of iBAT ANS activity, rats were euthanized by cardiac puncture. The gonadal fat mass was collected and weighed, representing the visceral fat deposit. BAT was dissected, weighed, and prepared for morphological studies or molecular measurement (kept at −80°C). The whole brain was removed and stored at −80°C until dissection of the nuclei of interest.

### BAT morphological analysis

BAT samples were fixed in formalin (freshly prepared in 1.27 M formaldehyde, 0.1 M phosphate-buffered saline, pH 7.2) and embedded in Paraplast Plus (Sigma-Aldrich, USA) for non-serial 5-µm thick sections. These sections were placed onto glass slides for hematoxylin/eosin staining and digital images were acquired randomly (TIFF format, 36-bit color, 1,360×1,024 pixels) using an Olympus DP71 camera and an Olympus BX40 epifluorescence microscope (Olympus, Japan). At least 10 photomicrographs per animal were randomly measured with the software Image-Pro Plus 5.0 (Media Cybernetics, USA). Photomicrographs were used for selection of fat droplets. The digital images of the droplets were analyzed and their areas calculated; the resulting data are shown in a histogram.

### Isolation of the hypothalamic nuclei

We used a cryostat (Hyrax C52, Zeiss, Germany) to obtain the coronal sections of the brain. The ARC (Bregma −1.6 to −2.6 mm), PVN (Bregma −1.8 to −2.1 mm), and VMH (Bregma −1.8 to −3.2 mm) were isolated in accordance with the coordinates described in the Paxinos and Watson stereotaxic atlas (25). The samples were frozen (at −80°C) for western blotting.

### Western blotting analysis

Protein content in the iBAT, PVN, and VMH was evaluated by western blotting. Briefly, BAT, PVN, and VMH were homogenized in RIPA buffer [50 mM Tris-HCl; pH 7.4, 1% NP-40, 150 mM NaCl, 1 mM EDTA, 1 mM PMSF, 1 mM Na_3_VO_4_, 1 mM NaF] and protease inhibitor cocktail (F. Hoffmann, La Roche Ltd., Switzerland). BAT was sonicated (three pulses of 5 s with 40% amplitude, intercalated by 15 s off). The homogenates were centrifuged three times (18,506 *g*, 4°C, 5 min) and the intermediate phase of the supernatant was collected after each centrifugation. At the end of the third centrifugation, the supernatant collected was adjusted to the final volume of 700 µL with RIPA buffer. The PVN and VMH were sonicated (two pulses of 10 s with 40% amplitude, intercalated by 15 s off). Protein concentration in the supernatants was determined using the Pierce BCA Protein Assay Kit (Thermo Scientific, USA). Then, homogenates were analyzed by SDS-PAGE using 30 mg total protein for BAT and 10 mg total protein for PVN. Both samples were transferred onto PVDF membranes (Hybond ECL; Amersham Pharmacia Biotech, UK). Membranes were incubated with Tris-buffered saline (TBS) containing 5% albumin for 45 min. Subsequently, the membranes were washed with TBS then incubated with specific primary antibodies: anti-UCP1 (1:500, Sigma-Aldrich, USA), anti-TRβ1, anti-TRα1 (1:500, Abcam, UK), anti-β3-AR (1:500, Santa Cruz Biotechnology, Inc., USA), anti-PGC1α, and CPT1a (1:1000, Santa Cruz Biotechnology, Inc.) overnight at 4°C. The same procedure was performed with the PVN and VMH membranes, which where incubated with anti-MC4R (1:500, Abcam) and anti-AMPKα or anti-phospho-AMPKα (1:500, Cell Signaling Technology, Inc., USA), respectively. The primary antibody anti-β−actin was used as internal control for each membrane (1:500, Sigma-Aldrich). Membranes were washed three times with Tween-TBS (0.1%) and then incubated for 1 h with the appropriate concentration of the secondary antibody (1:1000, 1:7000 or 1:1000) conjugated with biotin (anti-rabbit, anti-mouse or anti-goat Sigma-Aldrich) at room temperature. Then, the membranes were washed again three times with Tween-TBS (0.1%), which was followed by the incubation with streptavidin-conjugated horseradish peroxidase (Caltag Laboratories, USA). Protein bands were visualized by chemiluminescence (Kit ECL plus, Amersham Biosciences, UK) followed by exposure to ImageQuant LAS (GE Healthcare, UK). The area and density of the bands were quantified by Image J software (Wayne Rasband National Institute of Health, USA) and normalized against the bands obtained for β-actin. Results are reported as the relative percentage (%) of the control group (C).

### Reverse transcription polymerase chain reaction (RT-PCR) analysis

Tissues were previously stored in RNAlater (Qiagen, USA) at −80°C to avoid RNA degradation. Total RNA was extracted from the iBAT, under RNAse-free conditions, using the RNeasy¯ Lipid Tissue Mini Kit (Qiagen) in accordance with manufacturer’s recommendations. The quantity and the quality of the RNA samples were evaluated using the NanoVueTMPlus Spectrophotometer (GE Healthcare, England). Then, each RNA sample was diluted to obtain the final concentration of 1 µg/µL. Before the cDNA construction, RNA samples were treated with DNAse (RQ1 RNase-Free DNase- Promega, USA). The cDNA was constructed from the total RNA using the Moloney Murine Leukemia Virus Reverse Transcriptase (M-MLV RT) for RT-PCR and Oligo (dT) 15 Primer (Promega). The mRNA expression was analyzed by real-time RT-PCR carried out in triplicate for each sample using an Applied Biosystems 7500 Real-Time PCR System (Applied BioSystems, USA). In order to ensure no amplification of genomic DNA, we performed a minus RT reaction (RT-) in all real-time PCR assays and no amplification product (Cq value) was detected in any of the RT-control reactions. The mRNA level of Dio2 (Assay ID: Rn00581867_m1) expression was evaluated using TaqMan¯ Fast Universal PCR 11 Master Mix (2X) AmpErase¯ UNG (Catalog #4324018; Applied Biosystems¯, USA) according to the recommendations of the manufacturer. The co-amplification of β-actin gene (Assay ID: Rn00667869_m1) was performed as an internal control in all samples. This gene has been chosen as the reference gene since there were no statistical difference between the Cq mean of the control group and the smoke group. Assays were performed in triplicate and the results were analyzed using the ΔΔCT method.

### Immunohistochemistry

We used one male and one female per litter of both groups, (n=7/group) for immunohistochemistry analysis. They were anesthetized with 70 mg/kg body weight ketamine and 7 mg/kg body weight xylazine and intracardially perfused with 0.9% saline, followed by 4% paraformaldehyde (in phosphate buffer, pH 7.4), and then by the same fixative plus 10% sucrose. After dissection, brains were immersed in phosphate buffer containing 20% sucrose overnight at 4°C for cryoprotection. Specimens were then frozen and coronally sectioned in 20 µm sections in a cryotome at −20°C (Hyrax C52, Zeiss) in Tissue-Tek O.C.T. compound (Sakura). Sections containing the hypothalamus, specifically the ARC region (Bregma −1.6 to −2.6 mm), according to Paxinos and Watson (25), were collected in gelatinized slides and stored at −20°C. To perform the immunohistochemistry, slides were treated with 0.3% PBS-Triton X-100 followed by a blocking solution (5% bovine serum albumin from Sigma) for 1 h at room temperature. We proceeded with incubation with anti-POMC antibody (from Santa Cruz, produced in rabbit, diluted in 1% bovine serum albumin, 1:100) overnight at 4°C. Immunoreaction was visualized by the secondary antibody anti-rabbit conjugated with ALEXA FLUOR 488 (produced in donkey, diluted 1:400; Invitrogen, USA) for 1 h at room temperature. After rinses with PBS, slides were mounted in ProLong Gold antifading reagent with 40,6-diamidino-2-phenylindole (DAPI) (Invitrogen, Molecular Probes, USA). Omission of the primary antibodies with inclusion of the secondary antibody was also performed for the negative control procedure. Images were captured using an epifluorescence microscope (BX-40; Olympus, Japan). For the quantification of POMC, positive cells were counted in the captured images for both male and female analysis (four slices counterstained with DAPI per nucleus per animal).

### Statistical analysis

Data were analyzed with the statistical program GraphPad Prism 5.0 for Windows (GraphPad Software, USA) and reported as means±SE. First, each variable was analyzed by two-way ANOVA, with group (C *vs* SE) and gender (males *vs* females) as between-subject factors. If the initial analysis indicated both group and gender effects or interaction between these factors, then data were re-examined by one-way ANOVA followed by Newman-Keuls post-hoc test. On the other hand, if the initial analysis indicated only a group effect, data were analyzed using Student's unpaired *t*-tests, evaluating separately the developmental plasticity effects in males and females. Differences were considered significant at P<0.05 (Table 1).

**Table 1.** Long-term effects of neonatal cigarette smoke exposure on all analyzed parameters in 180 postnatal day offspring.

Parameters	Group effect	Gender effect	Interaction
Gonadal fat mass (g)	Yes (F = 8.76, P = 0.005)	No (F = 2.61, P = 0.114)	No (F = 0.57, P = 0.454)
iBAT SNA	Yes (F = 10.14, P = 0.004)	Yes (F = 11.91, P = 0.002)	Yes (F = 4.46, P = 0.047)
Lipid droplets (% area)	No (F = 0, P = 0.987)	Yes (F = 9.9, P = 0.005)	No (F = 0.14, P = 0.716)
UCP1 content	Yes (F = 12.47, P = 0.001)	No (F = 0.52, P = 0.478)	No (F = 0.14, P = 0.478)
β3-AR content	Yes (F = 5.99, P = 0.022)	No (F = 0.75, P = 0.397)	No (F = 0.75, P = 0.397)
TRα1 content	Yes (F = 4.91, P = 0.039)	No (F = 0.80, P = 0.381)	No (F = 0.54, P = 0.470)
TRβ1 content	Yes (F = 5.19, P = 0.034)	No (F = 1.36, P = 0.258)	No (F = 0.06, P = 0.816)
PGC1α content	Yes (F = 7.1, P = 0.013)	No (F = 0.35, P = 0.558)	No (F = 0.35, P = 0.558)
CPT1a content	Yes (F = 15.49, P = 0.0006)	No (F = 1.78, P = 0.194)	No (F = 1.78, P = 0.194)
Dio2 mRNA expression	No (F = 0, P = 0.980)	No (F = 0, P = 0.961)	No (F = 0.26, P = 0.615)
POMC (cell count)	Yes (F = 0, P = 0.984)	Yes (F = 37.12, P<0.0001)	No (F = 1.15, P = 0.317)
MC4R level	No (F = 0,61, P = 0.442)	No (F = 1.05, P = 0.316)	No (F = 1.05, P = 0.316)
AMPK content	No (F = 0.03, P = 0.863)	Yes (F = 8.22, P = 0.007)	Yes (F = 8.22, P = 0.007)
pAMPK content	Yes (F = 4.63, P = 0.041)	Yes (F = 14.97, P = 0.0007)	Yes (F = 14.97, P = 0.0007)

Total food intake represents the sum of the total food intake between postnatal day 21 and 180. Data are reported as means±SE, n=8/group. Comparisons were done with the control group using ANOVA. iBAT: interscapular brown adipose tissue; SNA: sympathetic nerve activity; UCP1: uncoupling protein 1; β3-AR: beta 3-adrenergic receptor; TRα1 and TRβ1: TH receptors; PGC1α: peroxisome proliferator-activated receptor-coactivator; CPT1a: carnitine palmitoyltransferase; Dio2: type 2 iodothyronine deiodinase; POMC: proopiomelanocortin; MC4R: melanocortin 4 receptor; AMPK: AMP-activated protein kinase; pAMPK: phosphorylated AMPK.

## Results

### Biometric parameters

As shown in Table 2, both genders of the SE group presented higher food intake during development compared to the respective controls (males: +7%; females: +6%, P<0.05), but only SE females had increased body mass at PND180 (+9% *vs* C, P<0.05). Regarding fat deposit, a group effect was observed (F_1,36_=8.76). The SE group showed higher gonadal fat mass (+41% *vs* C males; +55% *vs* C females, P<0.05). The iBAT mass also showed group (F_1,34_=12.18) and gender (F_1,34_=13.33) effects. The SE group had higher iBAT mass (+51% *vs* C males, +31% *vs* C females, P<0.05; +53% *vs* SE females, P<0.05).

**Table 2.** Long-term effects of neonatal cigarette smoke exposure on body mass, food intake, visceral fat mass, and iBAT mass in 180 postnatal day-offspring.

	Males		Females	
	C	SE	C	SE
Body mass (g)	451±14	462±13	249±7	272±8*
Total food intake (kg)	3.37±0.08	3.60±0.05*	2.34±0.03	2.48±0.20*
Gonadal fat mass (g)	4.7±0.4	6.7±0.7*	5.5±0.4	8.5±0.9*
iBAT mass (g)	0.24±0.03	0.36±0.03*	0.18±0.01	0.23±0.01*

C: control; SE: smoke-exposed; iBAT: interscapular brown adipose tissue. P<0.05, *t*-test.

### BAT SNA

**Figure 2.**
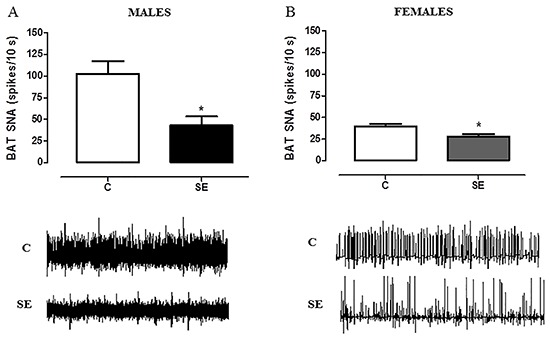
Long-term effects of neonatal cigarette smoke exposure on brown adipose tissue (BAT) sympathetic nerve activity (SNA). Number of spikes in 10 s at 180 postnatal days in males (*A*) and females (*B*). Representative recordings are shown at the bottom of the figures. C: control group; SE: smoke-exposed group. Data are reported as means±SE for n=6. *P<0.05 (two-way ANOVA re-examined by one-way ANOVA followed by Newman-Keuls post-hoc test).

An interaction was observed between group and gender (F_1,20_=4.46). Both genders showed group effects (F_1,20_=10.14, Figure 2), as the SE group showed lower SNA at the basal condition in BAT compared to its respective controls (−58% *vs* C males; −30% *vs* C females, P<0.05). The gender effect (F_1,20_=11.91, Figure 2) is explained by the lower BAT SNA in the controls (−61% in C females *vs* C males, P<0.05).

### BAT morphology and function

**Figure 3.**
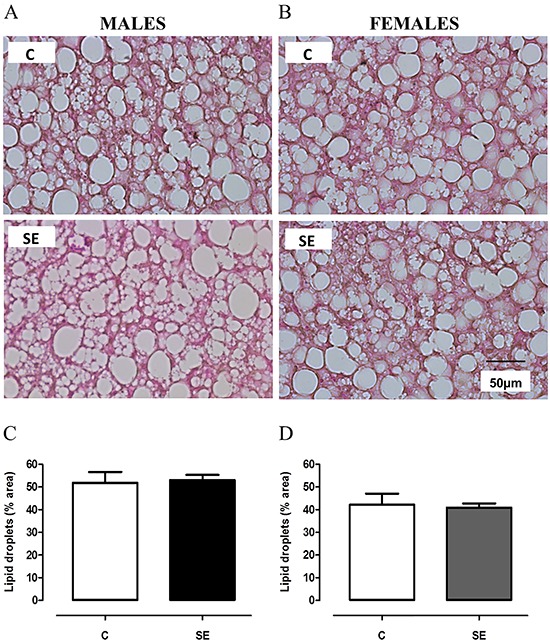
Long-term effects of neonatal cigarette smoke exposure on brown adipose tissue (BAT) morphology. Representative hematoxylin and eosin staining of BAT at 180 postnatal days in males (*A*) and females (*B*) (Scale bar: 50 µm). Quantitative analysis of the sectional areas of BAT lipid vacuoles is shown in males (*C*) and females (*D*). C: control group; SE: smoke-exposed group. Data are reported as means±SE for n=6. There was gender effect, but there was no group effect for the lipid droplet sectional area (two-way ANOVA).

As depicted in Figure 3, there was a gender effect regarding lipid droplet sectional area variable (F_1,21_=9.90): SE females had smaller areas compared with SE males (−23%, P<0.05). No group effect was observed.

Thermogenesis biomarkers showed neither interaction nor gender effects. Thus, only the group effect was considered for the variables shown below.

**Figure 4.**
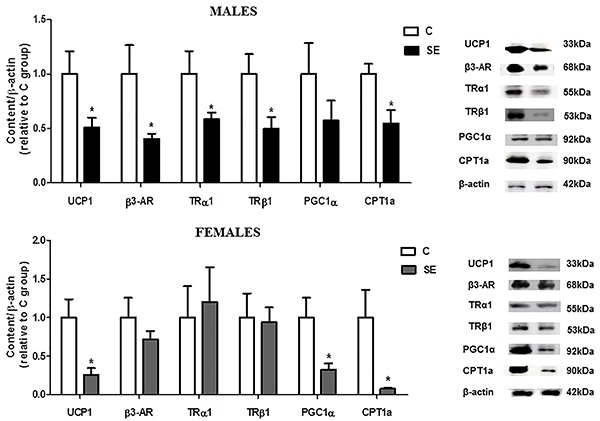
Long-term effects of neonatal cigarette smoke exposure on brown adipose tissue (BAT) functional parameters in male and female offspring. UCP1, β3-AR, TRα1, TRβ1, PGC1α, and CPT1a protein contents in BAT at 180 postnatal days. Representative blots of the proteins are shown beside the graphs. β-Actin content was used as control loading. C: control group; SE: smoke-exposed group. Data are reported as means±SE for n=7–8. *P<0.05 (*t*-test).

At PND180, both genders of the SE group showed lower UCP1 protein content (−49% *vs* C males; −74% *vs* C females, P<0.05) and CPT1a (−45% *vs* C males; −92% *vs* C females, P<0.05). Besides, male SE rats displayed lower protein content of β3-AR (−60% *vs* C), TRα1 (−42% *vs* C), and TRβ1 (−50% *vs* C) (P<0.05) while female SE rats showed lower protein PGC1α expression (−67% *vs* C females, P<0.05). The protein content of PGC1α was not altered in SE males, whereas in the SE females, β3-AR, TRα1, and TRβ1 protein contents were unchanged (Figure 4).

**Figure 5.**
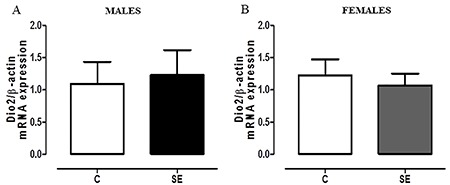
Long-term effects of neonatal cigarette smoke exposure on brown adipose tissue (BAT) Dio2 mRNA expression at 180 postnatal days in males (*A*) and females (*B*). C: control group; SE: smoke-exposed group. Data are reported as means±SE for n=6–8 (*t*-test).

As shown in Figure 5, both genders in the SE group had no change of Dio2 mRNA levels at adulthood. No effects or interaction were observed regarding Dio2 gene expression.

### Hypothalamic POMC, MC4R, and AMPK

**Figure 6.**
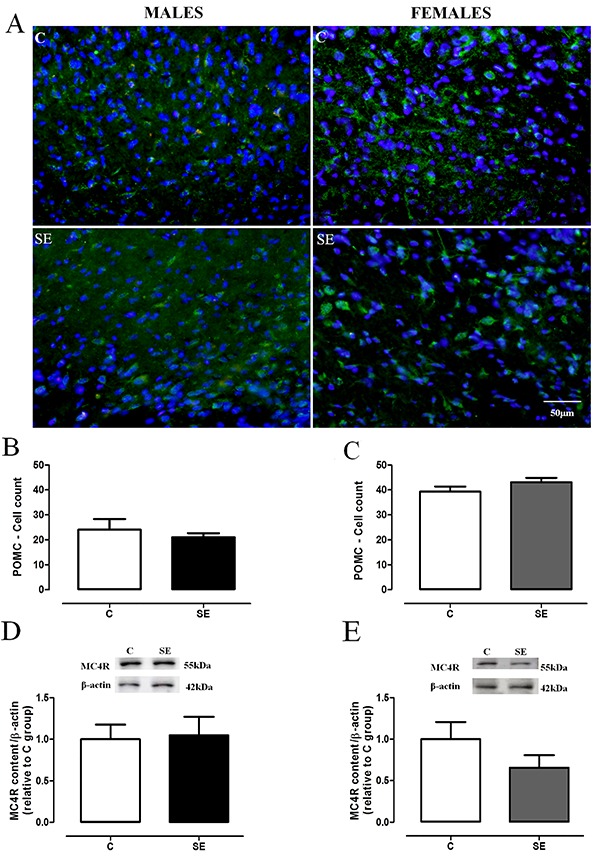
Long-term effects of neonatal cigarette smoke exposure on POMC and MC4R in the hypothalamus. Qualitative data of POMC (in green), counterstained for DAPI (in blue) in arcuate nucleus by immunohistochemistry (*A*) (Scale bar: 50 µm) and quantitative data concerning the number of POMC-positive cells in males (*B*) and females (*C*). Protein content of MC4R in the paraventricular nucleus (PVN) at 180 postnatal days in males (*D*) and females (*E*) with representative blots of proteins. β-Actin content was used as loading control. Data are reported as relative % to the control group. C: control group; SE: smoke-exposed group. Data are reported as means±SE for n=7 (two-way ANOVA re-examined by one-way ANOVA followed by Newman-Keuls post-hoc test).

At PND180, the POMC immunostaining in the ARC (Figure 6A-C) showed a gender effect (F_1,21_=37.12) in the controls (+100% in C females *vs* C males, P<0.05) as well as in the SE animals (+103% in SE females *vs* SE males, P<0.05).

The MC4R protein content in the PVN (Figure 6D and E) was similar between groups and genders. There was no effect or interaction regarding this variable.

**Figure 7.**
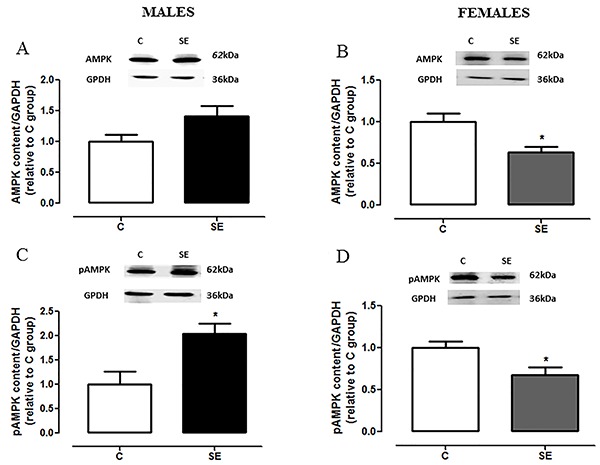
Long-term effects of neonatal cigarette smoke exposure on AMPK and pAMPK in the hypothalamus. AMPK (*A* and *B*) and pAMPK (*C* and *D*) protein contents in the ventromedial nucleus of the hypothalamus at 180 postnatal days in males and females. Representative blots of proteins are shown above the graphs. GAPDH content was used as loading control. Data are reported as relative % to the control group. C: control group; SE: smoke-exposed group. Results are reported as means±SE for n=6–8. *P<0.05 (*t*-test).

As for AMPK and pAMPK (depicted in Figure 7), interactions between factors (AMPK: F_1,29_=8.22 and pAMPK: F_1,24_=14.97) and gender effect (AMPK: F_1,29_=8.22 and pAMPK: F_1,24_=14.97) were observed. A group effect (pAMPK: F _1,24_=4.63) in protein content in the VMH was also identified. SE males showed increased pAMPK content (+104%, Figure 7C, P<0.05), whereas the SE females had decreased AMPK and pAMPK protein content in the VMH (−36 and −32%, Figure 7B and D, P<0.05).

## Discussion

In the present study, we have demonstrated that exposure to cigarette smoke exclusively during lactation was capable of causing BAT hypofunction in the adult offspring of both genders, reducing autonomic nerve activity and important markers related to thermogenesis. Since SE males had higher serum thyroid hormones (TH) (7–9), they are probably not functional in BAT. Thus, we suggest that neonatal tobacco smoke exposure programs males for TH resistance in BAT.

Although gender-related differences were not the primary focus of our evaluation, we found gender effects regarding BAT ANS only in control animals; however, no gender effect was observed on BAT biomarkers analyzed in both C and SE groups. In the literature, studies on thermogenesis and gender differences are scarce and still controversial (26–28). It has already been reported that estrogen can reduce BAT thermogenesis (26), which can explain the decrease in BAT ANS in control females compared to males.

Besides the obesity in both genders, which is in agreement with previous data (7–9), and the lower ANS in the basal condition demonstrated in the current study, postnatal cigarette smoke exposure led to an increase in iBAT weight in adult life without changing the percentage lipid droplet area in the brown adipocytes. Recently, Fan et al. (18) have demonstrated that nicotine exposure during gestation and lactation is also capable of altering iBAT function, causing whitening, reduction of functional mitochondria, and reduction in UCP1, PGC1α, CPT1a, and in other thermogenic-related markers. Here, male and female SE animals also showed reduced thermogenic biomarkers levels. Thus, although tobacco smoke contains thousands of substances, it is possible that nicotine plays the most important role in this programming effect. Fan et al. (18) found marked morphological changes that are possibly explained by the wider window of exposure, which encompassed both gestation and lactation. In the present study, most of the BAT adipogenesis was not influenced by smoke exposure, which might be explained by the fact that exposure in our study occurred only during lactation. It is possible that hypofunction precedes BAT whitening since another study has reported alterations in BAT function without morphological changes (29). The long-term effect of neonatal exposure to cigarette smoke on UCP1 is closely related to the reduction in thermogenic capacity and body fat gain in the SE group because UCP1 is a key molecule for the facultative thermogenesis (11,12).

The β3-AR in rodents is important for regulating BAT thermogenesis (30); therefore, the lower β3-AR content in the SE males contributes to reduce the sympathetic activity in these animals. However, no difference was observed in β3-AR content in the SE females. Despite this, the catecholamine signaling pathway can be disrupted in females.

Another mediator of BAT thermogenesis is CPT1, a limiting enzyme in the transport of free fatty acids into the mitochondria for the oxidation and heat generation processes (31). The lower content of this enzyme in the SE groups of both genders contributes to BAT malfunctioning and increased body fat in these animals since it is known that higher CPT1a activity is related to the increase in BAT thermogenesis, increasing its oxidation rate of fatty acids (31).

Other regulators of adrenergic signaling in BAT are the thyroid hormones (TH) (32). As previously reported, male SE animals show an increase in TSH and TH levels indicating central hyperthyroidism (7). Female SE had only increased serum T4 (9). Here, we demonstrated that SE males had lower TRβ1 and TRα1, indicating TH resistance in BAT, thus contributing to a lower thermogenic action and UCP1 expression. TRβ1 isoform stimulates the expression of UCP1 and TRα1 isoform increases the adrenergic activity in BAT (14). Thus, it seems that, at least in males, TH resistance compromises autonomic nerve activity in BAT.

In our study, BAT Dio2 mRNA expression was not altered in the SE group of both genders, despite the reduction of TH receptors in the SE males. Dio2 activity/expression is responsible for T4 to T3 conversion, ensuring the cytoplasmic T3 pool (15). It is well known that plasma TH negatively regulate this enzyme (15). As the SE group had increased serum TH, a lower BAT Dio2 expression was expected. Thus, the unchanged Dio2 found in our model also suggests TH resistance at BAT level.

BAT PGC1α is stimulated by both adrenergic tonus and TH levels (32). In SE females, the reduction of BAT PGC1α can contribute to the reduction in thermogenic activity, since PGC1α is a transcriptional co-activator that mediates events related to energy metabolism, capable of interfering in the expression UCP1 in the BAT (33), besides acting in the control of mitochondrial biogenesis (34). Since SE females had hyperthyroxinemia, we expected to find increased BAT PGC1α. Thus, again, this is suggestive of TH resistance at least in females.

The melanocortin system activation in the PVN controls BAT ANS activity (35,36). Despite the reduction of ANS activity in SE animals, the MC4R content in the PVN as well as in the number of POMC positive cells were unaltered in our model, suggesting that the BAT ANS dysfunction was not related to melanocortin system programming.

As previously published, SE males have higher TSH and TH with increased body fat (7). Thus, the hyperthyroid status of this group was not enough to cause body mass loss. It is known that T3 inhibits AMPK in the VMH and increases sympathetic nervous system activity (21). Thus, the increase in AMPK in the VMH of SE males is suggestive of TH resistance. Also, higher AMPK content explains hyperphagia, lower SNA, and lower thermogenic markers, since AMPK enhances food intake and decreases thermogenesis (20). SE males had lower SNA associated with decreased adrenergic and TH receptors in BAT, indicating that higher TH is also not functional in this tissue. Conversely, it seems that SE females have a normal response to T4 in the hypothalamus since AMPK content was reduced. Thus, SE females have a less intense reduction of BAT SNA compared with SE males (30 *vs* 58%). Other factors may be acting on SE females that may help explain the persistent lower BAT SNA, such as leptin resistance, as suggested in a previous study (9).

**Figure 8.**
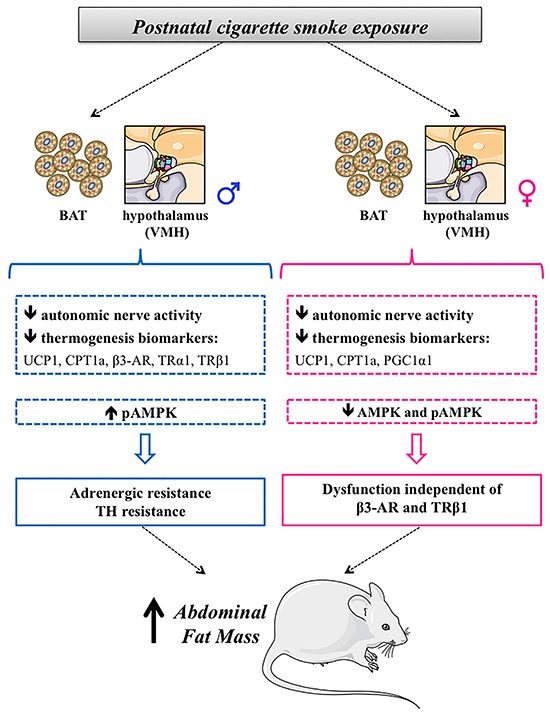
Smoking in critical windows of development causes epigenetic alterations and is a risk factor for adulthood obesity. BAT: brown adipose tissue; UCP1: uncoupling protein 1; β3-AR: beta 3-adrenergic receptor; TRα1 and TRβ1: thyroid hormones receptors α1 and β1; CPT1a: carnitine palmitoyltransferase 1a; PGC1α: peroxisome proliferator-activated receptor-coactivator; VMH: ventromedial nucleus of the hypothalamus; AMPK: AMP-activated protein kinase; pAMPK: phosphorylated AMPK; TH: thyroid hormone.

Figure 8 summarizes the main findings of the current study. Here we showed that tobacco smoke exposure during the suckling period reduced the thermogenic capacity of BAT in both genders, possibly explaining the increase in body adiposity in adulthood, independently of BAT melanocortin system regulation. Apparently, the reduction in BAT SNA stimulation was almost twice as intense in males. In males, the main mechanism for the observed alterations can be attributed to adrenergic and TH resistance in both BAT and VMH. In females, however, this resistance is not completely clear since it seems to be present only in BAT and it is not dependent on β3-AR and TRβ1 function.
